# Modulation of the Genome and Epigenome of Individuals Susceptible to Autism by Environmental Risk Factors

**DOI:** 10.3390/ijms16048699

**Published:** 2015-04-20

**Authors:** Costas Koufaris, Carolina Sismani

**Affiliations:** Department of Cytogenetics and Genomics, the Cyprus Institute of Neurology and Genetics, P.O. Box 3462, Nicosia 1683, Cyprus; E-Mail: costask@cing.ac.cy

**Keywords:** xenobiotic, immune, endocrine, epigenome, gut microbes, oxidative stress, transposable elements, endocrine disruptors, transgenerational, gene environment interactions, seizures, genotype

## Abstract

Diverse environmental factors have been implicated with the development of autism spectrum disorders (ASD). Genetic factors also underlie the differential vulnerability to environmental risk factors of susceptible individuals. Currently the way in which environmental risk factors interact with genetic factors to increase the incidence of ASD is not well understood. A greater understanding of the metabolic, cellular, and biochemical events involved in gene x environment interactions in ASD would have important implications for the prevention and possible treatment of the disorder. In this review we discuss various established and more alternative processes through which environmental factors implicated in ASD can modulate the genome and epigenome of genetically-susceptible individuals.

## 1. Introduction

Autism spectrum disorders (ASD) are a collection of neurodevelopmental disorders typically diagnosed in the first three years of life, characterized by deficits in verbal and nonverbal communication, repetitive behaviors, and impairments in social interactions [1]. ASD is caused by the genetic or epigenetic disruption of genes that are essential for normal neurodevelopment, with hundreds of candidate susceptibility genes identified so far. Genetic variations associated with ASD include large chromosomal abnormalities, copy number variants (CNV), indels/deletions, and single nucleotide variants (SNV). The genetic variants implicated in ASD are either inherited from parents to affected individuals or form *de novo* in the patients. Inherited genetic factors (both common and rare variants) have been estimated to explain ~40% of ASD risk [2], while *de novo* mutations are thought to contribute to 15%–20% of cases [3]. Recurrent mutations found in ASD patients implicate disruption of certain biological pathways as particularly important in ASD, such as post-synaptic density [4], phosphoinositide 3-kinase (PI3K)-mammalian target of rapamycin (mTOR) [5], and ubiquitin processing [6].

Strong evidence now also implicates epigenetic dysregulation with ASD development. For example, the epigenetic regulator methyl CpG binding protein 2 (*MECP2*) is well established as being implicated in ASD [7], while altered DNA and histone epigenetic markers have been identified in brain tissue and immune-cell derived cells from autistic patients [8,9,10,11]. The biological significance of the observed epigenetic alterations in ASD is supported by the observation that they affect the expression of genes implicated in ASD such as *MECP2*, retinoic acid related orphan receptor A (*RORA*), and engrailed-2 (*EN-2*) [8,9,10,11].

Of particular interest to both researchers and the general public is the substantial evidence linking exposure to environmental agents with the incidence of ASD. Epidemiological studies have associated diverse prenatal and perinatal environmental exposures to an increased risk of ASD [12,13,14], while discordance between monozygotic twins in the incidence of the disorder also supports the importance of environmental influences [15]. Additionally, the largest genetic study reported so far also indicated a large influence of environmental factors on the development of ASD [2]. A sensible hypothesis, based on the importance of both genetic and environmental factors on ASD, is that the interactions between these two components influence the incidence of the disorder. Indeed, a number of recent reviews have discussed the evidence from genetic, epidemiological, and animal studies that support the influence of gene x environment interactions on ASD [16,17,18,19]. However, our understanding of the biological mechanisms underlying genotype-environment interactions in ASD is currently inadequate.

## 2. Disruption of the Genome and Epigenome of Genetically Susceptible Individuals by ASD Environmental Risk Factors

Environmental ASD risk factors are those non-genetic factors that can influence the development of the disorder in genetically-susceptible individuals. These have been suggested to include dietary factors, maternal diabetes, pre-and-perinatal stress, parental age, medications, zinc deficiencies, supplements, pesticides, and infections. Ultimately the environmental risk factors act by disrupting the genome/epigenome of developing neurons. This review will not aim to provide an overview of the numerous proposed environmental ASD risk factors. Rather here we will focus on how environmental factors can modulate the genome and epigenome of individuals susceptible to develop ASD. An increased understanding of this gene-environment interaction would have great implications for the prevention and treatment of the disorder. For example, identifying genetically vulnerable individuals would allow targeted strategies for reducing their exposure to ASD risk factors. Taking into account genotype-environment interactions could also improve the consistency and interpretation of association and epidemiological studies, which often fail to replicate. Finally, elucidating the mechanisms of genotype-environment interactions could also inform the identification of novel therapeutic targets in ASD patients.

### 2.1. Defective Xenobiotic Metabolism of ASD Environmental Risk Factors

The initial interaction between environmental toxins and biological organisms involves xenobiotic metabolic enzymes, specialized systems responsible for detoxifying and removing harmful chemicals from the body. Although the liver is considered the primary organ where xenobiotic metabolism takes place, detoxification in the brain is also known to occur [20]. Xenobiotic metabolic enzymes are also involved with the biochemical processing of neurotoxicants and environmental ASD risk factors. For example, 2,2',4,4'-tetrabromodiphenyl ether (BDE-47), a main component of the flame retardant polybrominated diphenyl ethers (PBDE) linked to ASD [21], is metabolised to its more harmful metabolites by action of CYP2B6 [22]. It is also the case that several environmental risk factors associated with ASD risk are established mutagens, possibly acting by increasing the incidence of mutations in key neurodevelopmental genes [23]. The majority of mutagenic compounds have to be metabolically converted from non-mutagenic to mutagenic forms by the action of cytochrome p450 (CYP450) enzymes, thus are also strongly affected by the functioning of xenobiotic metabolic activity.

Xenobiotic enzymes display extensive variation in copy number between individuals and ethnic groups. Additionally, SNVs are also known to affect the activity of xenobiotic enzymes. Polymorphisms in xenobiotic enzymes are an important source of individual variability in the response to environmental agents. Indeed, such a mode of action is widely accepted to influence individual susceptibility to environmental carcinogens [24]. A reasonable hypothesis then is that genetic variability that affects the metabolism of environmental ASD risk factors can render subsets of the population particularly vulnerable to environmental chemicals that modulate the genome or epigenome. However, so far few studies have examined the association between polymorphisms affecting xenobiotics metabolic enzymes and ASD incidence. One interesting case involves paraoxonase (*PON1*), a gene involved in the removal from the body of organophosphate pesticides. D’Amelio *et al.* [25] reported that variants of the *PON1* gene were associated with increased incidence of ASD in USA populations, but not in Italian populations. The authors interpreted these observations as relating to the higher levels of organophosphate to which the USA population is exposed. A second study reported an association between *PON1* polymorphisms and neurodevelopment in children exposed in utero to organophosphate pesticides [26]. Five studies have examined the association between *PON1* polymorphisms and ASD. These studies have had mixed results, with only three out of the five studies finding an association between the genetic variants and the disorder [27].

A study by Serajee *et al.* [28] examined the association between polymorphisms affecting six xenobiotic metabolism genes and ASD in 196 families and reported a significant association for a polymorphism affecting the metal-regulatory transcription factor 1 (*MTF1*). Buyske *et al.* [29] examined the association between deletions of the glutathione *S*-transferase M1 (*GSTM1*) allele and autism. This enzyme is involved in the detoxification of electrophilic compounds. In this study a significant association was found for homozygous deletion of *GSTM1* and ASD risk. In conclusion, genetic variability affecting the functioning of xenobiotic enzymes can render individuals more sensitive to environmental ASD risk factors. Taking into account exposures to environmental factors and variants affecting xenobiotic enzymes might potentially improve the design and interpretation of epidemiological studies, as demonstrated by D’Amelio *et al.* [25].

### 2.2. Increased Sensitivity to Endocrine-Disrupting Chemicals

Increasing evidence indicates that defects in the physiological sex hormone-dependent processes can influence ASD development [30,31]. Diagnosis of ASD is four times more frequent in males compared to females, which might be partly attributed to hormonal involvement in the development of the disease. Fetal testosterone affects brain organization [32] and investigation of amniotic fluid found elevated levels of sex steroid hormones in boys diagnosed with autism [33]. Importantly for our purposes, diverse environmental agents (including plasticizers, flame retardants, drugs, and pesticides) are acknowledged to be able to block or mimic normal hormone signaling by affecting the synthesis of hormones or by interacting with their receptors. Such environmental agents, termed endocrine-disruptor chemicals (EDC), have been implicated with the development of diverse human diseases, including ASD [34]. In support of the influence of EDC on ASD, epidemiological studies have linked gestational exposure to EDC with behavioral problems relating to ASD development [35,36].

Vulnerability to EDC exposures is known to be highly dependent on individual genotype. For example, disruption of male reproductive development in juvenile mice following EDC exposure varied more than 16-fold between mouse strains [37]. Individual variability in sensitivity to EDC can arise by various means, for example different basal levels of sex hormones or binding affinity of hormone receptors. Genetic variants affecting the sex hormones signaling axis in the developing brain could render individuals more susceptible to low EDC doses that induce abnormal brain development and ASD. One study has reported an association between polymorphisms of the androgen receptor and ASD [38]. A second recent study examined the association between 29 single nucleotide polymorphisms (SNP) in genes relating to sex steroids and ASD-like traits belonging to a subset (*n* = 1771) from The Child and Adolescent Twin Study in Sweden (CATSS) [39]. The authors reported two SNPs to be associated with autism-like traits, one located in the 3'UTR of the estrogen receptor and the other a non-synonymous variant affecting the 3-oxo-5-α-steroid 4-dehydrogenase 2 (SRD5A2) enzyme that is involved in testosterone metabolism. Another interesting observation is the reduced expression of RORA, a gene involved in the transcriptional regulation of genes that convert male to female hormones, in the brain and lymphocytes of ASD patients [9]. Nevertheless there is inadequate understanding at present of how abnormal sex hormone signaling can disrupt normal neurodevelopment and neurobiology in order to contribute to ASD.

### 2.3. Increased Susceptibility to Agents that Induce Oxidative Stress

Oxidative stress refers to an imbalance between the generation and removal of reactive oxygen species (ROS) in biological systems. Accumulating evidence supports that oxidative stress is also implicated with the development of ASD [40,41,42]. ROS can directly attack and damage DNA, thus increasing the incidence of mutations that can potentially contribute to ASD. A second possible consequence of oxidative stress is destabilization of the epigenome. In conditions of oxidative stress the enhanced requirement for the antioxidant glutathione can impair synthesis of *S*-adenosyl methionine (SAM), the major methyl donor used for DNA methylation, thus diminishing the available pool of methyl donors used for methylating DNA. The subsequent loss of normal methylation patterns results in the loss of normal regulation and aberrant gene expression. In addition, DNA hypomethylation can also lead to genomic instability [43]. A third detrimental consequence of ROS is the induction of mitochondrial dysfunction, which can initiate vicious cycles of increased ROS generation from the damaged mitochondria. Mitochondrial dysfunction can affect biological functions of the organelle that are important in normal neuronal functioning, such as regulation of neurotransmission [44]. The brain is considered to be particularly vulnerable to ROS due to the fact that it contains non-replicating cells that can become permanently dysfunctional or undergo necrotic or apoptotic cell death.

Increased oxidative stress has been reported in autistic patients [45] and a more oxidized microenvironment is associated with a more favourable development [40]. Additionally, mitochondrial disease and markers of mitochondrial dysfunction are much higher in ASD population compared to control populations [41]. A recent study has also found decreased tryptophan metabolism in lymphoblastoid cells from ASD patients [42]. Aberrant tryptophan metabolism is important because this amino acid is the precursor of molecules that are important for mitochondrial energy generation and anti-oxidant defenses [46]. Tryptophan metabolism is involved in the generation of NAD^+^ involved in mitochondrial energy generation through oxidative phosphorylation. Tryptophan is also ultimately involved in the generation of melatonin, which acts as a scavenger of free radicals [46].

Importantly, diverse types of environmental factors can act to either augment or protect against oxidative stress. Subsets of individuals with inherently higher rates of ROS formation or reduced anti-oxidative capabilities would therefore be more vulnerable to environmental conditions that favor a pro-oxidant microenvironment that can contribute to ASD development. Recently published studies have reported that oxidative stress induces more prominent mitochondrial dysfunction in lymphoblastoid cells derived from subsets of ASD patients [47,48]. Damage in the mitochondria can also lead to even greater oxidative stress as ROS are released from the damaged mitochondria. In an interesting recent study it has been reported that exposure of lymphoblasts to bisphenol A results in greater levels of oxidative stress and mitochondrial dysfunction in ASD individuals compared to unaffected siblings [49].

At present the greatest effort to identify the underlying genetic basis for an increased vulnerability of ASD patients to environmental agents has been carried out in relation to ROS and mitochondrial dysfunction [40,41,42,45,47,48,49,42,45,47]. Work in animal models also supports that genes implicated with ASD affect ROS and mitochondria. Examples are conditional phosphatase and tensin homolog (*Pten*) haplo-insufficient mice which display aberrant social behavior coupled with mitochondrial dysfunction [50] and neuroligin knockout *Caenorhabditis elegans* that show both abnormal behavior and increased sensitivity to oxidative stress [51]. However, since these two proteins have multiple biological functions it is not possible to exclude that loss of these genes contributes to ASD development through alternative mechanisms. Association studies have revealed an increased frequency of genetic variants affecting Methylenetetrahydrofolate reductase (*MTHFR*) [52] and superoxide dismutase 1 (*SOD1*) [53], enzymes that affect cellular anti-oxidative machinery, in ASD patients. MTHFR is a crucial enzyme of the folate pathway and is involved in the generation of the anti-oxidant glutathione and in generating methyl units that are used for repairing genetic and epigenetic damage caused by ROS. Intriguingly, a SNP that results in reduced activity of the MTHFR was associated with greater incidence of autism, but only in countries with low levels of folate fortification during pregnancy [51]. SOD1 is a member of the superoxide dismutase family that are important participants in antioxidant defense mechanisms. Researchers have recently reported the presence of rare variants within the non-coding potentially regulatory regions of the *SOD1* gene. Although not verified experimentally, the authors consider a plausible hypothesis to be that these variants will affect the regulation of this gene and subsequently the ability of individuals to respond to oxidative stress [53].

A number of recent studies have also identified increased frequencies of genetic variants that affect mitochondrial functions in ASD patients. Smith *et al.* [54] found an increased presence of CNV affecting genes involved in mitochondrial oxidative phosphorylation. Nava *et al.* [55] identified mutations affecting trimethyllysine dioxygenase (*TMLHE*), an enzyme catalyzing the first step of carnitine biosynthesis, in ASD patients. Carnitine is a molecule that is essential for mitochondrial fatty acid metabolism, as well as having anti-oxidant functions. Dysfunction in the mitochondrial aspartate/glutamate carrier (*AGC1*) involved in calcium homeostasis has also been reported in autism [56]. Another alternative mechanism that could render individuals more sensitive to environmental inducers of oxidative stress is the presence of persistent inflammation and immune dysregulation. In these individuals the immune system would result in the continuous generation of excess ROS that will act to drain the available anti-oxidative defenses of cells, rendering them more vulnerable to environmental agents [57].

### 2.4. Susceptibility to Epigenomic Dysregulation

Epigenetic markers such as DNA methylation and histone modifications regulate gene expression in eukaryotic cells. The proper epigenetic marking of DNA is crucial for the normal development of human tissues and organs. Studies of ASD patients have found aberrant patterns of epigenetic regulation, both in the brain and in lymphoblastoid cells [8,9,10,11,58]. Aberrant epigenetic mechanisms can contribute to ASD development by causing abnormal gene expression patterns or due to increased genomic damage occurring subsequent to DNA hypomethylation. By their very nature epigenetic mechanisms are at the interface between the genome and environmental factors. A variety of environmental factors can affect the epigenome, such as exposure to heavy metals and levels of dietary folate. Genetic factors that influence the interaction between environmental factors and the epigenome could therefore be important in determining the risk of ASD developing in exposed individuals. In one interesting study it was shown that mice with a truncated version of *Mecp2*, a regulator of the epigenome in neuronal cells, demonstrated social behavioral defects when exposed prenatally to the organic pollutant PBDE. Decreased sociability was associated with reduced global DNA methylation in the female but not in the male mice [21]. As mentioned previously, SNPs that reduce activity of MTHFR have been associated with increased risk of ASD in countries with low fortification [52]. One of the effects of reduced MTHFR activity is lower generation of methyl groups by the folate cycle that can be used for methylating DNA. An increased propensity towards a dysregulated epigenome could also arise due to an inherently higher baseline level of ROS, which use one-carbon units for the generation of glutathione, in ASD patients (discussed in Section 2.3).

An alternative mechanism that would render individuals more susceptible to environmental modulators of the epigenome has been proposed by La Salle [16]. According to this hypothesis large-scale genomic differences between individuals in the size of their repetitive regions can affect their vulnerability to environmental factors that influence the epigenome. It is being increasingly recognized that large-scale genomic differences exist between individuals. Polymorphic regions in the human region include duplications, deletions, inversions, microsatellite repeats, ribosomal DNA repeats, and interstitial telomeric repeats. Since the repetitive regions of the genome act as major drains of methyl donors, it is suggested that individuals with greater genomic size of repetitive elements will be more vulnerable to environmental factors that interact with the DNA methylome [16].

### 2.5. Hyperactive Transposable Elements

Transposable elements are DNA sequences that can mobilize themselves to new regions of the genome, thus acting as mutagens. Long interspersed element-1 (L1) is the most abundant autonomous transposable element in the human genome. Importantly, the L1 retrotransposition is active in neural progenitor cells [59]. Additionally, L1 retrotransposition is enhanced by a large variety of chemical treatments [60]. Intriguingly, reduced activity of Mecp2, a gene implicated in Rett syndrome and ASD, is associated with a higher occurrence of L1 retrotransposition [61]. Therefore, a propensity to a hyperactive mobility of L1 elements in neurons in response to environmental exposures is a plausible mechanism that can drive the incidence of the pathogenic mutations that in turn drive the development of the disease. The precise mechanisms by which environmental agents activate L1 retrotransposition are diverse and probably differ between environmental agents. For example, heterocyclic amines activate L1 mobility through activation of ligand-bound transcription factors [62] and alcohol affecting methylation [63]. As of yet, clear experimental evidence for an increased susceptibility to L1 retrotransposition hyperactivity caused by environmental agents in ASD remains to be reported.

### 2.6. Increased Genomic Instability

Genomic rearrangements lead to the formation of CNV through deletions, duplications, inversions, and complex rearrangements. Importantly, genes involved in neurodevelopmental disorders including ASD are often flanked by segmental duplications or repetitive elements which drive genomic rearrangements and disruption of gene function [64]. An interesting observation is that ASD patients possess significantly elevated CNV load, even when removing rare and pathogenic events [65]. This finding suggests that beyond the sampling bias this population is also characterized by an increased rate of genomic rearrangements [65]. Environmental agents such as ionizing radiation and genotoxic chemicals are known to be able to initiate genomic rearrangements which subsequently lead to CNV formation. An increased genomic instability in individuals following exposure to such environmental agents could contribute to ASD by enhancing the rate of genomic rearrangements that involve genes implicated in the disorder. Increased genomic instability could be the result of defects in the machinery involved in DNA repair and recombination [65]. So far one study has suggested a link between defective DNA repair and ASD development. Fanconi-associated nuclease 1 (*FAN1*) is a relatively recently identified nuclease involved in the repair of DNA inter-strand cross-links. In a recent study rare variants affecting the FAN1 have been suggested to be candidate drivers of ASD [66]. A decreased ability to repair DNA damage caused by environmental agents could also arise due to the presence in the genome of susceptible individuals of regions with significant sequence homology that flank genes important for normal neurodevelopment. Such genomic regions can act as substrates to allow improper chromosomal recombination (non-allelic homologous recombination or microhomology-mediated break-induced replication) to occur, resulting in loss or gain of the intervening genes.

### 2.7. Abnormal Immune Activation

Several studies have reported abnormalities in the peripheral immune system of ASD individuals and a number of genes that are frequently mutated in ASD relate to immune function [67]. Additionally recent epidemiological reports link infections in early pregnancy to an increased incidence of autism [12]. Increased neuronal inflammation is also observed in the central nervous system of ASD patients [67]. Consequently, it is firmly established that the immune system is dysregulated in autism and it has been suggested that this could be a contributing factor to the development of the disorder [67,68]. Cytokines receptors are expressed in the brain and cytokines released by immune cells are able to affect both the development and function of the neuronal system. Imbalanced cytokine release can therefore disrupt physiological neurodevelopment [69]. Prenatal maternal inflammation can also disrupt global and gene-specific methylation patterns in the brain [70]. Chronic inflammation can also be a source of ROS contributing to oxidative stress [40,41] which has been implicated in ASD (see Section 2.3).

Environmental exposures that trigger immune responses include infections, toxins that impact the immune system, and allergens. An increased propensity to immune dysregulation predisposing to ASD could be due to inappropriate activation of immune responses, prolonged and persistent immune responses, and autoimmunity [67,68]. Abnormal immune activation can also occur due to defective clearance of environmental toxicants by xenobiotic metabolic enzymes. In one interesting study Ashwood *et al.* [71] showed that peripheral blood mononuclear cells from control and autistic individuals resulted in qualitatively different profiles of secreted cytokines following bacterial lipopolysaccharide (LPS) exposure when pre-treated with environmental toxins PBDEs. This experiment suggested an inherently different immune response of ASD individuals to PBDE exposure compared to controls. Evidence in support of genotype-environment interactions for the immune system is also available from animal studies. Mouse research has shown a link between maternal immune activation and ASD behavior [67]. In a recent interesting study comparing two mice strains, C57BL/6J and Black and Tan BRachyury *T + tf/*J (BTBR), it was shown that the immune responses and their propensity for the development of ASD-like phenotypes in the offspring differed following exposure to viral mimics, with the BTBR strain displaying a greater susceptibility [72]. Consequently, this study supports the importance of gene x environment interactions in the development of immune system dysregulation and ASD. The genetic factors associated with the greater susceptibility of the BTBR strain were not clarified in this study, although Disrupted in Schizophrenia 1 (*Disc1*), a gene that this strain lacks and is involved in immunological responses, has been suggested as a potential candidate. In another recent study researchers showed that haploinsufficiency of tuberous sclerosis complex (*TSC*), a gene for which mutations are associated with a greatly increased risk of ASD development, interacted with infection to disrupt social behavior in adult mice [73]. In this case specific gene x environment interactions and their effects on normal behavior, at least in adult mice, were demonstrated.

### 2.8. Gut Microbiota

Gut microbiota are the collection of thousands of microbial species that are found within the intestine. The gut microbiota affects multiple biological systems such as gut permeability, immune system, and metabolism. Gut microbiota can also affect neuronal development, functioning, and signaling by interacting with the immune system, neural, and hormonal pathways [74]. As discussed in Section 2.7 abnormal immune system activation has been associated with ASD development. The gut microbiota also induce the generation of ROS in epithelial cells which can contribute to immune dysfunction and to oxidative stress [75]. Studies have reported differences in the gut microbiota between ASD patients and control cohorts [76,77]. In an intriguing recent study it was reported that in a mouse model of ASD altering the gut microbiota ameliorates behavioral abnormalities [78]. Importantly the gut microbiome is highly flexible and is affected by a person’s age, geographic region, environment, health, and genotype [79]. Studies in mice support that the genotype of animals is a strong determinant of the type of microbial species present in the animals guts [80,81]. Differences in the composition of the gut microbiota in humans could therefore potentially render them more susceptible to ASD environmental factors through distinct mechanisms e.g., increased sensitivity to environmental agents that disrupt the immune system. Currently we are not aware of any published studies that have investigated whether an individual’s gut microbiota can render them more or less susceptible to environmental risk factors for autism.

### 2.9. Transgenerational Environmental Effects

The transmission of the effects of environmental exposures across generations has been suggested to be involved in various human diseases, including autism and other behavioral disorders [82,83]. There is considerable debate about the mechanisms by which transgenerational information can be transmitted, although miRNA, DNA methylation, and histone modification are considered as primary candidates. Although highly speculative at present, it is possible that defects in epigenetic machinery could render individuals more susceptible to transgenerational epigenetic defects caused by environmental exposures. In one study gestational exposure to bisphenol A was linked to transgenerational behavioral abnormalities in mice [82]. A second study found that exposure to valproic acid *in utero* was associated with abnormal gut microbiome across multiple generation, as well as with immune and neurological abnormalities [83,84]. By causing transgenerational epigenetic effects, environmental factors could interact with genetic factors to promote ASD in individuals that were not exposed to the environmental agents directly.

### 2.10. Exacerbation of Environmental Effects by Seizures

Seizures are the result of abnormal electrical activity in the brain and can result in loss of consciousness and involuntary muscle contractions. Importantly, there is evidence for a link between seizures and ASD. There is a much higher prevalence of epilepsy in ASD children compared to controls [85]. Seizures are also associated with the exacerbation of ASD clinical features [86,87]. The interaction between seizures and autism could be important for two different reasons. First, seizures can modulate the incidence and clinical features of ASD associated with a given genotype. Second, seizures could render individuals more susceptible to environmental agents that disrupt normal neurodevelopment to promote ASD development. One proposed mechanism by which seizures can affect ASD is the downregulation of the Fragile X mental retardation protein (*FMRP*). It has long been known that Fragile X syndrome (FXS) is associated with increased incidence of ASD [88]. In recent years post-mortem examination of brain tissue from idiopathic ASD patients has also found deregulated FMRP [89,90]. Interesting research has found that seizures disrupt the normal functioning of the FMRP. In a rat model of seizures it has been found that this results in abnormal FMRP phosphorylation, localization, and function of the protein [91].

## 3. Conclusions

**Figure 1:**
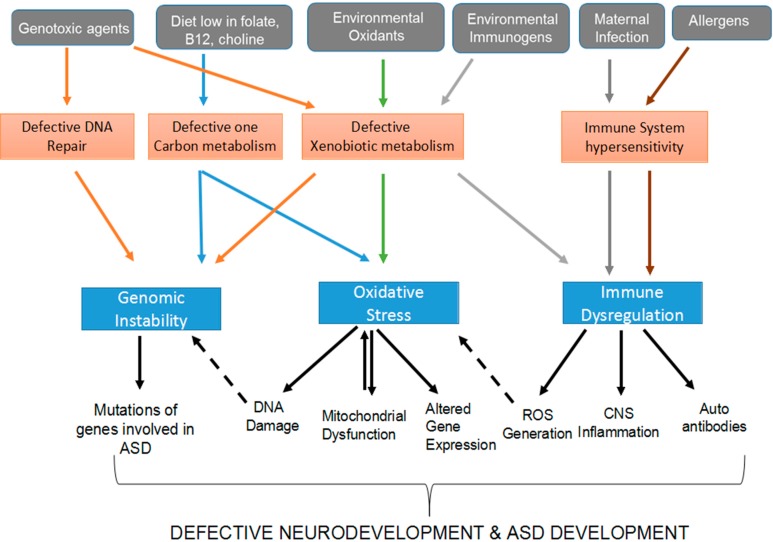
The figure illustrates the complex interactions between environmental and genetic factors that modulate the genome and epigenome of susceptible individuals. Environmental factors are shown in grey boxes, genetic factors in orange boxes, and the biological mechanism in blue boxes. The biological mechanisms shown in this figure are abnormal immune activation, oxidative stress, and genomic instability. The solid, colored arrows indicate interactions between environmental and genetic factors that eventually contribute to genomic instability, oxidative stress or immune activation. Orange colored lines indicate the effects of genotoxic agents, blue lines the effects of diets that are low in sources of one-carbon units, green lines indicate environmental agents, light gray lines indicate environmental immunogens, dark gray maternal infection, and red lines indicate allergens. Solid black lines link genomic instability, oxidative stress, and immune dysregulation to processes which contribute to ASD development. Dashed lines indicate interactions between biological processes involved in ASD e.g., DNA damage caused by oxidative stress also contributes to genomic instability and ROS generated by the immune system contribute to oxidative stress. As can be seen not only are multiple genotype-environmental interactions implicated in modulating the genome and epigenome of susceptible individuals, but interactions also occur between the different biological mechanisms.

So far substantially more research effort has been invested into cataloging the presence of genetic and epigenetic aberrations in ASD individuals compared to how and why these emerge in the first place. We have presented here a number of processes through which environmental ASD risk factors can modulate the genome and epigenome of genetically-susceptible individuals (Table 1). Some of these mechanisms have currently more supportive evidence (for example oxidative stress and immune dysregulation) while others are more speculative (for example transgenerational inheritance). Crucially, these different mechanisms do not act in isolation but can interact to enhance their detrimental effects. In Figure 1 we present examples of how different genetic and environmental factors and biological processes can interact to produce the genetic and epigenetic aberrations that promote ASD development. For example we have previously discussed the potential contribution of oxidative stress to ASD (Section 2.3). Defective one-carbon metabolism or dysfunctional mitochondria can increase the susceptibility of individuals to pro-oxidant diet or toxin exposures. Aberrant immune responses can also contribute to oxidative stress through the generation of ROS as a consequence of inflammation. Altered gut microbiota which disrupt the immune system could also possibly contribute to the generation of a state of oxidative stress. An integrative analysis of the multiple mechanisms of gene x environment interactions will be required to understand the development of ASD.

The susceptibility of individuals to environmental ASD risk factors is restricted to the early stages of life, especially during embryonic and fetal life, when the developing brain is uniquely sensitive to environmental agents [92,93]. The biological mechanisms described here should therefore only be relevant to ASD if they affect normal neurodevelopment *in utero* or possibly during very early post-natal stages. This observation raises the possibility that the genetically-susceptible individuals on which environmental risk factors act may not be the ASD patients themselves but their parents. For example an increased mutation rate as a consequence of environmental exposures (due to an inherently greater genomic instability or oxidative stress) could act to increase the presence of pathogenic mutations in the gametes and offspring. In this case mutations occurring in somatic tissues post-natally will not be relevant to ASD development. For many of the proposed mechanisms it is not clear at present whether environmental factors act on the ASD patients themselves or on their parents, a question of potential clinical significance.

It is important to remember when interpreting studies examining the association between exposure to environmental agents and ASD development that correlation does not necessarily imply causation. This is especially the case when the mechanisms through which environmental factors increase ASD risk are of a more speculative nature. Moreover, it is possible that environmental agents associated with ASD act to increase the incidence of the disorder through different mechanisms than the ones considered to be the primary candidates. Finally, in cases where environmental agents may act through multiple distinct mechanisms it can be difficult to determine which are the most relevant. For example, in conditions of low dietary folate MTHFR variants that affect the activity of the enzyme could increase the incidence of ASD by affecting oxidative stress, the epigenome or mutation rates (or all three). Uncovering the implicated mechanism could be of great therapeutic importance since epigenetic aberrations, unlike genetic aberrations, are in principle reversible.

**Table 1:** Mechanisms modulating the genome and epigenome of susceptible individuals.

Mechanisms	Effects on Genome and Epigenome	Environmental Factors	Genetic Factors
Oxidative stress	DNA damage; Mitochondria dysfunction; Disruption of the epigenome; Deregulated gene expression	Pro-oxidant toxicants; Diet low in one-carbon donors; Immunogens	Defective one-carbon metabolism; Defective anti-oxidant defenses; Abnormal immune activation; mitochondrial dysfunction
Abnormal immune activation	Autoantibody generation; CNS inflammation; Cytokine secretion; Gut permeability	Infections; Allergens; Immunogens	Defective xenobiotic metabolism; Autoimmune diseases; Hypersensitivity to immunogens
Genomic instability	Increased incidence of mutations predisposing to autism	DNA damaging agents; Diet low in one-carbon donors	Defective DNA repair; Defective xenobiotic metabolism; Genomic architecture
Epigenome dysregulation	Loss of normal gene regulation; Genomic instability	Heavy metals and toxins; CNS inflammation; Diet low in one-carbon donors	Defective one-carbon metabolism; Genomic architecture
Altered gut microbiome	Gut permeability; Neurotransmitter release; Immune dysfunction	Infections; Diet composition	Interaction of immune system with microbiota
Hyperactive transposable elements	Mutations affecting genes implicated in ASD	Chemicals activating retrotransposition	Reduced Mecp2 activity
Transgenerational inheritance	Abnormal gene expression patterns during neurodevelopment in unexposed generations	Perinatal stress; Endocrine disruptors; Effects on gut microbiome	Mechanisms are currently controversial

Elucidating the relevance of the candidate mechanisms by which environmental agents affect the genome and epigenome will require the usage of diverse research strategies. Family and population association studies that specifically examine genotype-environment interactions can provide strong evidence for the importance of such mechanisms in the disorder and identify genetic factors that increase individual susceptibility. We have previously discussed how variants affecting *MTHFR* have been associated with ASD risk according to folate fortification [52]. Another recent study reported that variants in the *MET* gene interact with air pollution exposure to increase ASD risk [94]. However, association studies examining genotype-environment interactions have low statistical power, necessitating very large study populations. Consequently, such studies are difficult to set up. A second limitation is that even when statistically significant gene x environmental factor interactions are detected by association studies, it can still be difficult to pinpoint the biological mechanisms by which these occur.

Highly useful for studies investigating the mechanisms of genotype-environment interactions in ASD are the repositories of biomaterials and phenotypic and genotypic data for ASD patients and unaffected relatives, such as the AGRE and SFARI depositories. Patient-derived lymphoblastoid cells can be highly informative as gene expression profiles of immune cells reflect to a surprising extent those of human tissues, including the brain [95]. Additionally, the utilization of induced pluripotent stem cells (iPSC) is becoming an increasingly more powerful methodology for simulating neurodevelopmental disorders in patients. The lymphoblastoid and iPSC from ASD patients and controls can be used to investigate differences in the vulnerabilities of ASD patients to environmental factors. Cell line based investigations can be performed much cheaper and quicker than association studies that require the recruitment of large numbers of patients. An additional advantage is that cell lines can be easily genetically manipulated, something that can be used for examining the biological mechanisms by which environmental agents interact with genetic factors. An example of a recent study using this approach to investigate a potential inherent susceptibility of autistic individuals was conducted by Main *et al.*, 2013 [96]. In this study the researchers compared the effects of treatment with hydrogen peroxide and *S*-nitroprusside on lymphoblastoid cell lines from six ASD-normal sibling pairs. They reported finding that the autistic individuals were more sensitive to necrotic death but not to DNA damage induced by this treatments. Although innovative this study was limited in its use of a very small sample size and in not examining the epigenetic susceptibilities of the cell lines derived from ASD individuals (for example by determining disruption of global and gene specific patterns of DNA and histone methylation).

A big limitation of *in vitro* methods is that they are not applicable to the study of neurodevelopmental or behavioural processes. Consequently a variety of animal models have been developed for use in ASD research, with different advantages and disadvantages. Lower order species such as *C. elegans* and zebrafish can be genetically manipulated relatively quickly and at a lower cost, while using mammalian models is trickier and more expensive, but can better replicate human neurobiology and complex social behavior. Animal models also provide powerful tools for investigating genotype X environment interactions in ASD. For example, studies have demonstrated that neuroligin-deficient *C. elegans* are more vulnerable to oxidative stress [51] and hippocampal-slices from Mecp2 mice are more sensitive to hypoxia [97]. A large number of animal models for ASD have already been developed, which can be used in the future for studies investigating gene X environment interactions.

An increased understanding of genotype-environment interactions in ASD is important for identifying key genetic variants and environmental risk factors, reducing the exposure of vulnerable individuals to environmental risk factors, increasing the power and efficacy of epidemiological and clinical traits, and could facilitate the development of personalized treatments. A priority of autism research in the coming years should therefore be to increase the investment of time and resources into elucidating the nature, extent, and importance of genotype-environment interactions in autism. This will require the systematic application of research approaches that will facilitate progress in the field of genotype-environment interactions in ASD.
